# Transcriptome-Based Treatment for Melanoma With Brain Metastasis: A Case Report

**DOI:** 10.7759/cureus.56494

**Published:** 2024-03-19

**Authors:** Mohamad Ammar Ayass, Kristen Melendez, Natalya Griko, Jin Zhang, Lina Abi-Mosleh

**Affiliations:** 1 Respiratory Medicine/Internal Medicine, Pulmonology, Nephrology, Ayass Bioscience LLC, Frisco, USA; 2 Respiratory Medicine/Internal Medicine, Pulmonology, Nephrology, Ayass Lung Clinic, Frisco, USA; 3 Bioinformatics, Ayass Bioscience LLC, Frisco, USA; 4 Research, Ayass Bioscience LLC, Frisco, USA

**Keywords:** case report, transcriptome, brain metastasis, melanoma, cancer

## Abstract

Malignant melanoma with brain metastasis has a high mortality rate. New approaches for diagnosis and treatment are urgently required to improve prognosis. Here we present a 60-year-old male with metastatic melanoma to the brain. Using a transcriptomics pipeline, we analyzed whole blood and resected tumor tissue, identifying enriched gene expression biomarkers and pathways - including seven upregulated ( BRAF, CDK4, EIF1AX, IK, NRAS, PIK3R2, and TP53) and 11 downregulated (CASP8, CDK10, CDKN2A, CTLA4, GNA11, HERC2, IRF4, MC1R, PLA2G6, RREB1, and TPCN2) genes in the blood (across 15 pathways), showing 14% enrichment, and 16 upregulated (CCND1, CDK4, CTLA4, EIF1AX, IK, IRF4, MITF, NRAS, PIK3CB, PIK3R2, PMEL, RREB1, SLC45A2, SOX10, TYR, and TYRP1) and three downregulated ( GNA11, KITLG, and PLA2G6) genes in tissue (across 17 pathways), showing 33% enrichment, with five shared markers and 12 shared pathways. The model connected CDK4 pathway overactivity observed in both samples to inhibitors like ribociclib, abemaciclib, and palbociclib as putative treatments. By enabling objective personalized therapy selection, this approach shows great promise for advancing patient outcomes.

## Introduction

Melanoma stands as a formidable skin cancer, contributing to the majority of skin cancer deaths. According to the American Cancer Society, projections for melanoma in the United States for 2024 estimate approximately 99700 noninvasive cases and 100640 invasive cases, with an anticipated 8290 deaths [1]. Among the oncogenic genes, *BRAF *is one of the most frequently mutated oncogenes found in melanoma, sparking the activation of the BRAF/MEK/ERK (MAPK) signaling pathway. Melanomas with BRAF mutations exhibit heightened aggressiveness compared to those with wild-type BRAF, often showing a greater tendency to metastasize to the brain, while metastasis to the brain is the primary cause of death in melanoma, occurring in 95% of cases [2]. Brain metastasis typically manifests relatively late in the disease course (stage IV), with a median interval of 2.2-3.8 years following the initial diagnosis [3].

Despite available treatment modalities such as immunotherapy, chemotherapy, surgery, and radiation therapy, the management of melanoma with brain metastasis remains challenging, with a median survival rate of less than one year [4]. The blood-brain barrier severely restricts the penetration of circulating therapies into brain tissue, posing major treatment obstacles for metastatic melanoma. Unfortunately, the prognosis for patients with advanced melanoma brain metastases has seen little improvement over the past 30 years. Once melanoma returns and spreads to the central nervous system, therapeutic options become extremely limited. Partaking in clinical trials or pioneering innovative treatment pathways based on emerging science may represent the only remaining beacons of hope for these patients with late-stage disease. Their outlook underscores the urgent need for continued research into novel CNS-based therapies that can overcome the blood-brain barrier and at last move the needle on outcomes for this terminal manifestation of melanoma.

In recent years, transcriptome studies have emerged as a valuable tool in cancer detection and treatment. The transcriptome represents the complete set of ribonucleic acid (RNA) molecules expressed by a cell, offering insights into various cellular states, gene function, genome plasticity, gene expression regulation, and individual transcript modification. Transcriptome analysis holds promise for enhancing the objectivity of melanoma diagnosis and prognostication, ultimately leading to improved patient outcomes. Recent efforts, such as by the Cancer Genome Atlas, have extensively characterized somatic mutations and their impact on the transcriptome in melanoma [5]. Transcriptome analysis has revealed pathways associated with melanoma metastasis, such as KDELR1 deficiency promoting metastasis and KDELR3 regulation suppressing metastasis [6]. Additionally, studies conducted by Raskin et al. have identified HMGA2 as a biomarker of melanoma progression and prognosis [7].

Building on these insights, our institution has developed a comprehensive transcriptome-based model. This model integrates the transcriptome profile, cytokine panel, and pharmaceutical database to identify gene expression differences, biomarkers, enriched or downregulated pathways, and potential therapeutic matches. By using this systems biology approach, we aim to enable personalized and precise treatment for patients. Assessing transcriptomic changes in routine blood tests shows immense potential for unlocking disease signaling dynamics in real-time. Our comprehensive transcriptome-based analysis tool is an innovative gene expression profiling panel analyzing over 18,000 RNA transcripts in whole blood. By detecting nuanced immune pathway alterations, the test serves as an informative liquid biopsy able to characterize the intricate tumor-microenvironment interaction. This systems biology approach reveals activated networks driving disease progression along with predicting optimal therapy matches and clinical trial eligibility from a standard blood draw.

Here, we present a case of a 60-year-old male diagnosed with malignant melanoma and brain metastasis (BRAF widetype). By comprehensively comparing the whole blood and brain tumor tissue transcriptome profiles, we endeavor to elucidate mechanisms underlying melanoma progression and immune evasion using the transcriptome model. This understanding seeks to open avenues for novel diagnostic strategies and targeted therapies, ultimately providing this patient with additional hope.

## Case presentation

The patient is a 60-year-old male with a history of stage 3 metachronous melanomas initially diagnosed in 2019. He had a local excision of basal cell carcinoma on the right cheek on 6/2019. No treatment was given after the resection. He had another local excision for melanoma on the right scapular with negative lymph nodes (Breslow score of 3, Breslow depth of 2.38 mm, AJCC T3a N1c M0). No additional treatment was given before or after the resection. A year after the initial diagnosis of melanoma, he developed multifocal metastatic nodules including the back. Subsequent resections show positive lymph nodes on 06/2020. Chemotherapy was administrated after resection, following immunotherapy with nivolumab and ipilimumab (beginning on 06/2020). Ipilimumab was discontinued after completing all four doses. Nivolumab was administered for nine cycles but also stopped in 2021 due to adverse reactions. In early 2023, the patient developed neurological symptoms including seizures, confusion, and memory loss. MRI revealed an enlarging left parietal lobe brain mass consistent with metastatic disease. He underwent resection of two left parietal melanoma brain metastases measuring 1.7 cm and 3.3 cm, respectively. Pathology showed BRAF wild-type disease. His chronic medications include atorvastatin 40 mg daily, metformin 500 mg twice daily, naltrexone 4.5 mg daily, and mebendazole 112 mg daily for three days on four days off each week. Following brain radiation, he was on dexamethasone, tapered off in March 2023. He also takes levetiracetam 500 mg twice daily and hydrocortisone (10 mg AM/5 mg PM) for adrenal insufficiency. Given this history of advanced melanoma with brain metastasis status post-resection and previous immunotherapy exposure, the patient has been referred by his oncologist for additional immunological assessment and prognostic considerations regarding subsequent treatment options.

In April 2023, his complete blood cell (CBC) count showed a decreased red blood cell (RBC) count of 3.98 x10^7^/μL (normal range: 4.63-6.08x10^7^/μL), and decreased hemoglobin (13.2 g/dL, normal range: 13.7-17.5 g/dL), while platelet and white cell count were within the normal range. Immunology antibodies were all negative. Complement C3 and C4 were within normal range. Additional labs showed elevated coagulation factor VIII activity (249.1%, normal range: 50-150%), decreased factor X activity (78.7%, normal range: 81.7-146.8%), and elevated D-dimer (434 ng/mL, normal range: 0-230 ng/mL). It also showed decreased lgG (550 mg/dL, normal range: 761-1560 mg/dL), lgA (64.3 mg/dL, normal range: 82-453 mg/dL), lgM (33.4 mg/dL, normal range: 46-304 mg/dL), and elevated apolipoprotein A1 (178 mg/dL, normal range: 90-170 mg/dL) (Table 1). Physical examination showed no abnormalities. No lesion was observed. The patient subsequently received IVIG therapy to benefit immune globulin deficiency. The patient had radiation therapy in May 2023.

**Table 1:** The patient blood test results RBC, red blood cell

Date	Test name	Test result	Normal range
04/2023	RBC count	3.98x10^7^/uL	4.63-6.08x10^7^/uL
04/2023	Hemoglobin	13.2 g/dL	13.7-17.5 g/dL
04/2023	Platelet count	233x10^3^/uL	163-337x10^3^/uL
04/2023	White cell count	6.41x10^4^/uL	4.23-9.07x10^4^/uL
04/2023	Complement C3	89.5 mg/dL	79-152 mg/dL
04/2023	Complement C4	20 mg/dL	16-38 mg/dL
04/2023	aPS/PT lgG	5.84 units	≤30 unit: negative; >30 unit: positive
04/2023	aPS/PT lgM	6.59 units	≤30 unit: Negative; >30 unit: positive
04/2023	C1qCIC	<1.23 ug eq/mL	<4.4 ug eq/mL: negative; 4.4-10.8 ug eq/mL: equivocal; ≥10.8 ug eq/mL: positive
04/2023	Anti-histone	0.63 units	<1.0 units: negative; 1.0-1.5 units: weak positive; 1.6-2.5 units: moderate positive; >2.5 units: strong positive
04/2023	Anti-ribosome P	0.42 units	<20 units: negative; 20-39 units: weak positive; 40-80 units: moderate positive; >80 units: strong positive
04/2023	Anti-chromatin	6.20 units	<20 units: negative; 20-60 units: moderate positive; >60 units: strong positive
04/2023	Dsdna antibodies	<0.5 IU/ml	<10 IU/mL: negative; 10-15 IU/mL: equivocal; >15 IU/mL: positive
04/2023	Smith D(P) antibodies	<0.8 EliA U/mL	<7 EliA U/mL: negative; 7-10 EliA U/mL: equivocal; >10 EliA U/mL: positive
04/2023	U1rnp antibodies	1.0 U/mL	<5 U/mL: negative; 5-10 U/mL: equivocal; >10 U/mL: positive
04/2023	Scl-70 antibodies	<0.6 U/mL	<7 U/mL: negative; 7-10 U/mL: equivocal; >10 U/mL: positive
04/2023	Jo-1 antibodies	<0.3 U/mlL	<7 U/mL: negative; 7-10 U/mL: equivocal; >10 U/mL: positive
04/2023	Anti-centromere	<0.4 U/mL	<7 U/mL: negative; 7-10 U/mL: equivocal; >10 U/mL: positive
04/2023	Ssa (Ro) antibodies	<0.3 EliA U/mL	<7 EliA U/mL: negative; 7-10 EliA U/mL: equivocal; >10 EliA U/mL: positive
04/2023	Rnp-70 antibodies	0.5 U/mL	<7 U/mL: negative; 7-10 U/mL: equivocal; >10 U/mL: positive
04/2023	Ssb (La) antibodies	<0.3 EliA U/mL	<7 EliA U/mL: negative; 7-10 EliA U/mL: equivocal; >10 EliA U/mL: positive
04/2023	Anti-Ccp	0.4 EliA U/mL	<7 EliA U/mL: negative; 7-10 EliA U/mL: equivocal; >10 EliA U/mL: positive
04/2023	Rheumatoid factor lgA	<0.6 IU/mL	<14 IU/mL: negative; 14-20 IU/mL: equivocal; >20 IU/mL: positive
04/2023	Rheumatoid factor lgM	<0.5 IU/mL	<3.5 IU/mL: negative; 3.5-5 IU/mL: equivocal; >5 IU/mL: positive
04/2023	Cardiolipin antibodies lgG	<0.5 GPL-U/mL	<10 GPL-U/mL: negative; 10-40 GPL-U/mL: weak positive; >40 GPL-U/mL: positive
04/2023	Cardiolipin antibodies lgA	0.6 APL-U/mL	<14 APL-U/mL: negative; 14-20 APL-U/mL: Equivocal; >20 APL-U/mL: positive
04/2023	Cardiolipin antibodies lgM	<0.8 MPL-U/mL	<10 MPL-U/mL: negative; 10-40 MPL-U/mL: weak positive; >40 MPL-U/mL: positive
04/2023	Beta 2 glycoprotein lgG	0.8 U/mL	<7 U/mL: negative; 7-10 U/mL: Equivocal; >10 U/mL: positive
04/2023	Beta 2 glycoprotein lgM	<2.9 U/mL	<7 U/mL: negative; 7-10 U/mL: Equivocal; >10 U/mL: positive
04/2023	Beta 2 glycoprotein lgA	0.6 U/mL	<7 U/mL: negative; 7-10 U/mL: Equivocal; >10 U/mL: positive
04/2023	Mpo - myeloperoxidase antibody	<0.3 U/mL	<3.5 U/mL: negative; 3.5-5.0 U/mL: equivocal; >5.0 U/mL: positive
04/2023	Pr3 - proteinase-3 antibody	<0.7 U/mL	<2.0 U/mL: negative; 2.0-3.0 U/mL: equivocal; >3.0 U/mL: positive
04/2023	GBM antibodies	<1.9 U/mL	<7 U/mL: negative; 7-10 U/mL: equivocal; >10 U/mL: positive
04/2023	Coagulation factor VIII	249.1%	50-150%
04/2023	Coagulation factor X	78.7%	81.7-146.8%
04/2023	D-dimer	434 ng/mL	0-230 ng/mlL
04/2023	lgG	550 mg/dL	761-1560 mg/dL
04/2023	lgA	64.3 mg/dL	82-453 mg/dL
04/2023	lgM	33.4 mg/dL	46-304 mg/dL
04/2023	Apolipoprotein A1	178 mg/dL	90-170 mg/dL
07/2023	RBC count	4.61x10^7^/uL	4.63-6.08 x10^7^/uL
07/2023	Hemoglobin	15.2 g/dL	13.7-17.5 g/dL
07/2023	Myoglobin	13.8 ng/mL	17.4-105.7 ng/mL
07/2023	Progesterone	0.04 ng/mL	0.1-2.1 ng/mL
07/2023	D-dimer	<200 ng/mL	0-230 ng/mL
07/2023	Coagulation factor VIII	220.7%	50-150%
07/2023	Coagulation factor X	98.1%	81.7-146.8%
07/2023	lgG	692 mg/dL	761-1560 mg/dL
07/2023	lgA	78 mg/dL	82-453 mg/dL
07/2023	lgM	55.5 mg/dL	46-304 mg/dL
07/2023	Apolipoprotein A1	173 mg/dL	90-170 mg/dL

In July 2023, lab tests showed that his CBC showed improved RBC (4.61x10^7^/μL), decreased myoglobin of 13.8 ng/mL (normal range: 17.4-105.7 ng/mL), and decreased progesterone (0.04 ng/mL, normal range: 0.1-2.1 ng/mL). Hemoglobin, D-dimer, and factor X activity were back within the normal range (Table 1). Levetiracetam was reduced from 500 mg twice a day to 250 mg once/day due to no seizure activity so far. MRI showed a left frontal brain tumor consistent with metastatic malignant melanoma. The patient had a left frontal brain tumor resection in August 2023. The pathology report showed that both submitted lesions showed extensive metastatic malignant melanoma measuring 0.3 cm in size and 1.1 cm in size, respectively. As follows, he had radiation therapy in September 2023 and October 2023.

In November 2023, a repeat brain MRI showed a slight increase in the size of the enhancing left lateral frontal mass consistent with malignant melanoma, now measuring 1.5x1.7 cm. There was also an enlarged peripherally enhancing mass measuring 2.2x1.5 cm with increased hemosiderin staining from the prior scan, consistent with recurrent/residual tumor. Moderate surrounding vasogenic edema was again noted, which had progressed compared to the previous study.

As outlined previously, the patient presents with stage IV melanoma characterized by multiple brain metastasis. Despite undergoing immune checkpoint therapy, surgery resections, and radiation therapies, the melanoma has recurred. Patients at this advanced stage face a very high mortality rate, with the median survival rate six months following diagnosis (high frequency of brain metastases after adjuvant therapy for high-risk melanoma). Given the dire prognosis associated with this case, there is an urgent need for innovative treatment strategies to combat recurrences. In pursuit of deeper biological insights and improved treatment strategies, we leveraged our transcriptomics platform on both peripheral blood (collected on 01/2024) and brain tumor samples from this patient (collected during the initial brain surgery on 02/2023). The subsequent section will provide a detailed overview of the findings from the two transcriptome profiles.

Whole blood and tissue RNA transcriptome sequencing analysis

Transcriptome sequencing analysis was performed on whole blood (collected on 01/2024) and formalin-fixed paraffin-embedded (FFPE) brain tumor tissue (left parietal collected on 02/2023). Total RNA from whole blood was extracted using standard protocols to isolate high-quality RNA from venous blood. For RNA extraction from the FFPE sample, the RecoverALL Total Nucleic Acid Isolation kit (ThermoFisher) was utilized. The total RNA extracted from the whole blood and FFPE tissue was used to prepare sequencing libraries using the Ion AmpliSeq Transcriptome Human Gene Expression Kit. This kit contains gene-specific primers that target over 18000 human genes. The sequencing was performed using the ThermoFisher Ion Torrent GENEXUS instrument. The purpose of this sequencing was to identify genes that showed significant expression differences between the patient and healthy controls. The transcriptome RNA sequencing data was then processed using ThermoFisher’s AmpliseqRNA workflow to generate the gene expression counts for each sample from the resulting BAM files from sequencing. The count data for the sample of interest was then compared to healthy controls using DESeq2 via the PyDESeq2 package for Python [8,9]. The comparison process involved normalization via the median of ratios. Genes that showed differential expression, otherwise known as differentially expressed genes (DEGs) were considered significant biomarkers for further analysis.

To characterize disease activity, we compare the identified DEGs to a library of biomarkers associated with the specific disease. The resulting score is an expression of how strong the activity is for that specific disease based on the DEGs identified in the sample. Diseases that show a score greater than 10% are likely to be present in the patient. In order to identify gene networks and hub genes, the positive and negative DEGs were separately analyzed using STRING. STRING helped identify protein-protein interactions and networks [10]. Hub genes were defined as genes that exhibited a higher degree of connectivity relative to other genes in the network. These hub genes were considered significant due to their high connectivity, suggesting they play a vital role in the interrelation between the DEGs. Further analysis involved conducting STRING analysis on the identified hub genes to produce the enriched pathways, including KEGG pathways. To quantify the enrichment, a strength score for each pathway is calculated by dividing the number of DEGs that are part of the pathway by the pathway’s total number of background genes. The enriched pathways and DEGs were then compared to a database of FDA-approved drugs extracted from KEGG to generate a list of pathway-related medications [11-13]. The comparison was done by first identifying all drugs in the database that are associated with the enriched pathways, then further filtered by the DEGs that are present in the sample. Additionally, a proprietary Ayass Bioscience, LLC database was used to provide a list of holistic treatments by comparing the enriched pathways and DEGs.

**Figure 1:**
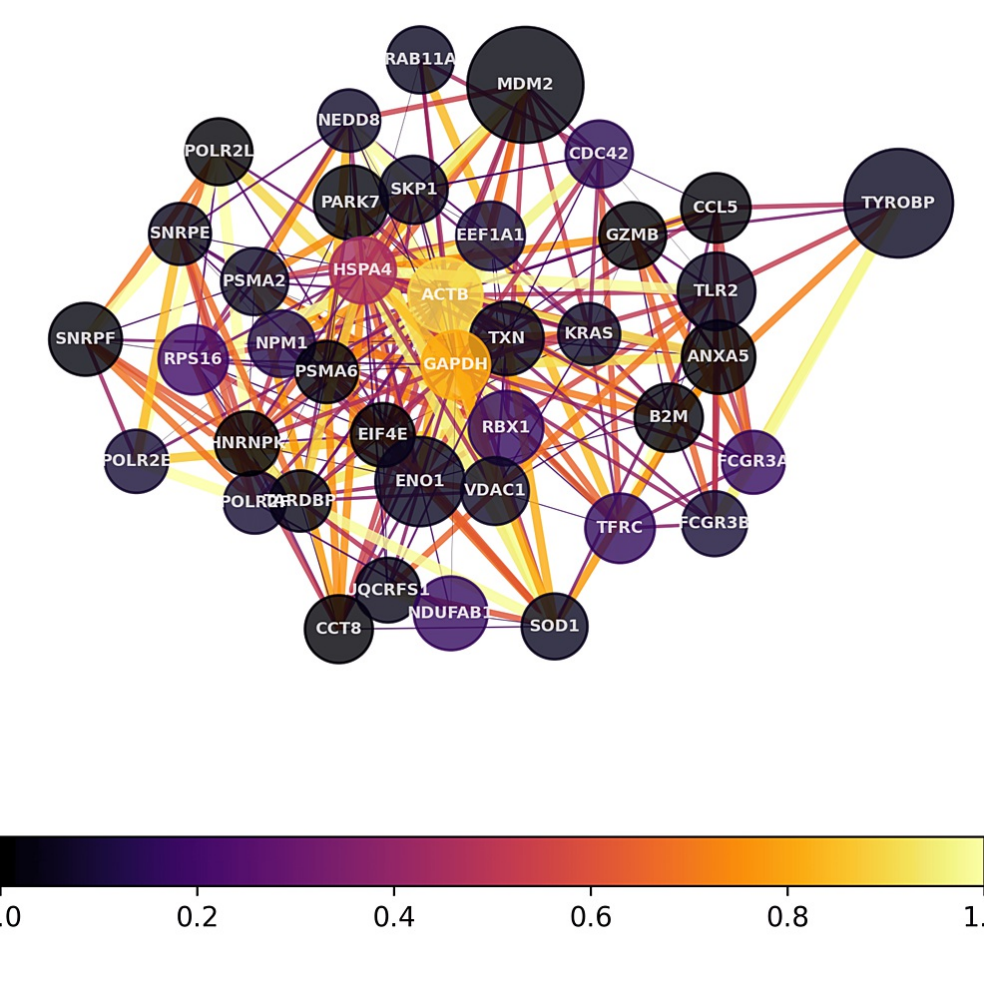
Hub gene network for the blood sample The hub gene network depicts the most highly interconnected genes identified in transcriptomic profiling of the patient sample. Hub genes were determined by analyzing differential gene expression and protein-protein interactions. These highly influential hub genes displayed more numerous and tighter connections, indicating they serve crucial communication roles within biological pathways disrupted in disease.

**Figure 2:**
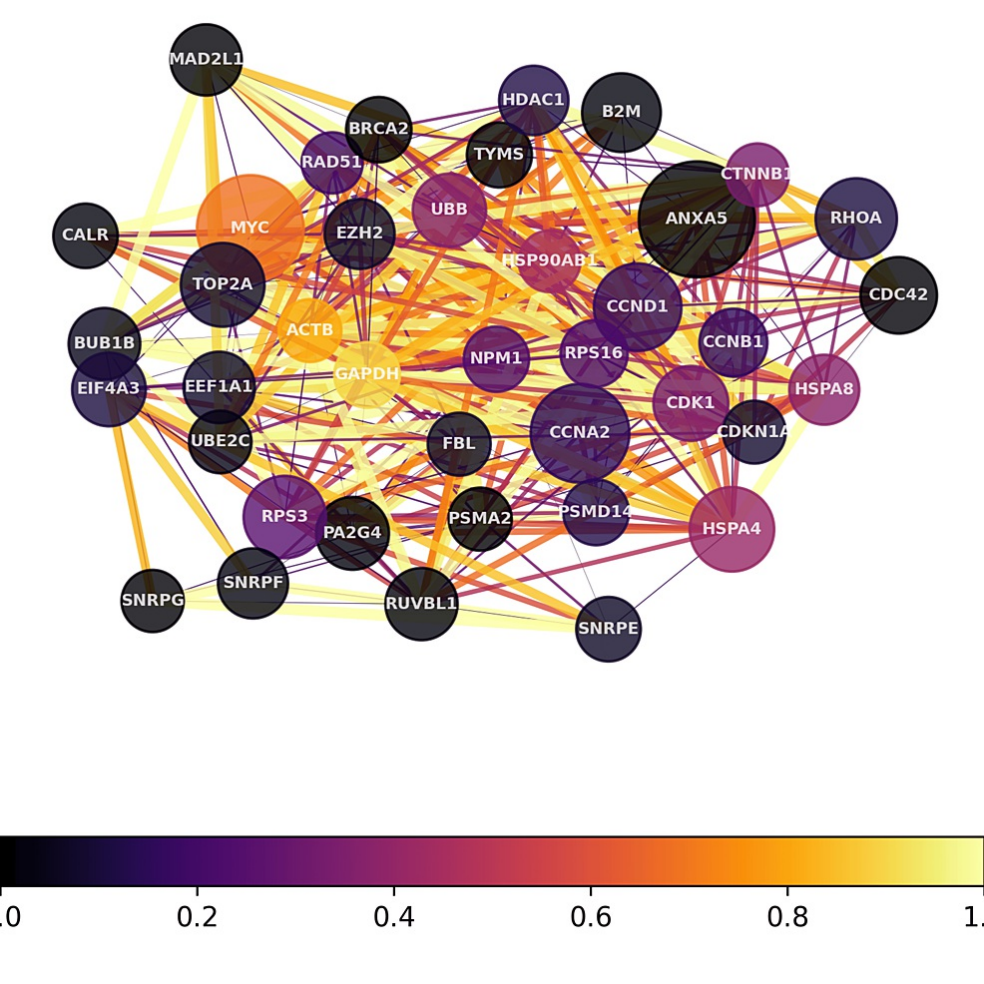
Hub gene network for the tissue sample The hub gene network depicts the most highly interconnected genes identified in transcriptomic profiling of the patient sample. Hub genes were determined by analyzing differential gene expression and protein-protein interactions. These highly influential hub genes displayed more numerous and tighter connections, indicating they serve crucial communication roles within biological pathways disrupted in disease.

Analysis using our proprietary transcriptome pipeline detected melanoma signals on both samples. For the whole blood, the transcriptome analysis revealed melanoma-related genes, seven of which were upregulated (BRAF, CDK4, EIF1AX, IK, NRAS, PIK3R2, and TP53) and 11 of which were downregulated (CASP8, CDK10, CDKN2A, CTLA4, GNA11, HERC2, IRF4, MC1R, PLA2G6, RREB1, and TPCN2), showing 14% enrichment for characterization of melanoma (Figure 1). For the brain tissue, the transcriptome analysis revealed 16 upregulated melanoma-related genes ( CCND1, CDK4, CTLA4, EIF1AX, IK, IRF4, MITF, NRAS, PIK3CB, PIK3R2, PMEL, RREB1, SLC45A2, SOX10, TYR, and TYRP1) and three downregulated genes (GNA11, KITLG, and PLA2G6) showing 33% enrichment (Figure 2).

At the same time, pathway analysis identified 15 and 17 enriched pathways in the whole blood sample (Table 2) and brain tissue (Table 3), respectively. Analysis of brain tissue also revealed two pathways that were downregulated (Table 4).

**Table 2:** Disease characterization: enriched melanoma pathways in a whole blood sample

ID	Pathway	Category	FDR	Activity score
hsa04666	Fc gamma R phagocytosis	Immune system	0.0	0.17
hsa04370	VEGF pathway	Cytokines and growth factors	0.0	0.16
hsa04210	Apoptosis	Cell growth and death	0.0	0.14
hsa05218	Melanoma	Disease pathway	0.005	0.11
hsa04660	T-cell receptor pathway	Immune system	0.001	0.11
hsa04664	Fc epsilon RI pathway	Immune system	0.01	0.11
hsa04662	B-cell receptor pathway	Immune system	0.008	0.1
hsa04670	Leukocyte migration	Immune system	0.006	0.09
hsa04810	Actin regulation	Cell motility	0.001	0.08
hsa04530	Tight junction	Cellular community	0.007	0.08
hsa04015	Rap 1 signaling	Signaling cascades	0.003	0.07
hsa04510	Focal adhesion	Cellular community	0.006	0.07
hsa04014	RAS/RAF/MEK pathway	Signaling cascades	0.004	0.07
hsa05200	PD-L1 pathway	Immune system	0.017	0.05
hsa05200	Pathway in cancer	Cancers overview	0.017	0.05

**Table 3:** Disease characterization: enriched melanoma pathways in brain tissue sample

ID	Pathway	Category	FDR	Activity score
hsa04520	Adherens junction	Celluar community	0.0	0.14
hsa04660	T-cell receptor pathway	Immune system	0.0	0.12
hsa05218	Melanoma	Disease pathway	0.001	0.11
hsa04670	Leukocyte migration	Immune system	0.001	0.09
hsa04370	VEGF pathway	Cytokines and growth factors	0.028	0.09
hsa04210	Apoptosis	Cell growth and death	0.001	0.08
hsa04530	Tight junction	Cellular community	0.0	0.08
hsa04658	Immune checkpoint pathways	Immune system	0.012	0.08
hsa04664	Fc epsilon RI pathway	Immune system	0.046	0.08
hsa04810	Actin regulation	Cell motility	0.002	0.07
hsa04510	Focal adhesion	Cellular community	0.002	0.07
hsa04015	Rap1 signaling	Signaling cascades	0.008	0.06
hsa05200	PD-L1 pathway	Immune system	0.0	0.06
hsa05200	Pathways in cancer	Cancers overview	0.0	0.06
hsa04151	PI3K-Akt pathway	Signaling cascades	0.004	0.05

**Table 4:** Disease characterization: downregulated melanoma pathways in brain tissue sample

ID	Pathway	Category	FDR	Activity score
hsa04010	MAPK pathway	Signaling cascades	0.0	0.17
hsa04014	RAS/RAF/MEK pathway	Signaling cascades	0.003	0.15

Overall, while certain signals that emerged were tissue-specific, our integrated pathway analysis revealed shared mechanisms across both sample types, indicative of interconnected systemic and local immune processes underlying disease advancement. Integrated analysis of the blood and tissue transcriptomic data revealed five biomarkers commonly enriched across both sample types, CDK4, NRAS, EIF1AX, IKZF1, and PIK3R2. Further examination of these shared genes and their known roles in melanoma biology provides important insights: 1) CDK4: The CDK4 gene encodes a protein that regulates cell division. Amplification or overexpression of CDK4 is found in a subset of melanomas and is associated with increased cell proliferation and tumor growth. 2) NRAS: The NRAS gene encodes a small GTPase protein involved in cellular signaling. Activating mutations in NRAS are found in 15-30% of melanomas and drive uncontrolled cell proliferation and survival. Mutant NRAS is a driver mutation in melanoma. 3) EIF1AX: The EIF1AX gene encodes a protein involved in protein synthesis. Recurrent mutations in EIF1AX were recently discovered in uveal melanoma, which is a rare type of melanoma found in the eye. These mutations appear to drive uveal melanoma development. 4) IK: This likely refers to the IKZF1 gene, which encodes the Ikaros zinc finger protein involved in hematopoietic cell development and regulation. 5) PIK3R2: The PIK3R2 gene encodes one of the regulatory subunits of PI3-kinase involved in cell growth signaling. Loss of function mutations in PIK3R2 has been observed in some cases of uveal melanoma, suggesting PIK3R2 acts as a tumor suppressor in the eye’s melanocytes. Further analysis revealed significant overlap at the pathway level as well, with 12 enriched pathways shared commonly between the whole blood and metastatic brain tissue transcriptomes: 1) T-cell receptor signaling pathway, 2) melanoma, 3) leukocyte migration, 4) VEGF signaling pathway, 5) apoptosis, 6) tight junction, 7) Fc epsilon RI pathway, 8) actin regulation pathway, 9) focal adhesion pathway, 10) Rap1 signaling pathway, 11) PD-L1 pathway, and 12) pathways in cancer. Additional correlation between cytokine and identified pathways revealed elevated cytokine levels of platelet-derived growth factor (PDGF), a signaling molecule known to be associated with various types of cancer, including melanoma. PDGF plays a vital role in regulating cellular processes such as cell growth, proliferation, and migration, which are often dysregulated in cancer cells. The software effectively identified and extracted several key pathways linked to PDGF and melanoma progression. These included the VEGF signaling pathway, which is crucial for promoting angiogenesis, enabling tumor growth and metastasis; focal adhesion signaling cascade, which regulates cell adhesion and migration, facilitating melanoma cell invasion and dissemination; Rap1 signaling pathway, known to modulate cell adhesion, migration, and invasion, was also identified as a relevant pathway. The integration of these pathways provides a comprehensive understanding of the molecular mechanisms underlying melanoma development and progression, by PDGF dysregulation.

Suggested pathway-related treatment

In both brain tumor tissue and whole blood, the system found elevation of CDK4 and associated pathways suitable for treatment with CDK4/6 inhibitor, ribociclib, abemaciclib, and palbociclib. Before releasing this case report, the patient in this case has yet to receive CDK4/6 inhibitors due to health insurance coverage and clinical protocols.

## Discussion

Melanoma, though comprising only 1% of all skin cancer, accounts for a striking 80% of skin cancer-related deaths [14]. While transcriptome sequencing analysis has been widely employed in research settings, its clinical application remains limited. Previously, we have reported a case of utilizing a transcriptome-based model to diagnose polycythemia vera and acute myeloid leukemia (under review). Here, we present a case utilizing the transcriptome model in the context of melanoma with metastasis to the brain. A unique aspect of this case lies in the acquisition of both whole blood and tumor tissue samples, allowing for integrated multi-omic analysis. Through the transcriptome analysis, we were able to discern unique gene expressions, biomarkers, and pathways relevant to melanoma, providing direct insight into disease pathogenesis.

Notably, the comparison of two transcriptome profiles revealed five common biomarkers and 12 pathways, providing compelling evidence of correlation. Both whole blood and tumor tissue exhibited elevated levels of CDK4, suggesting a targeted treatment approach utilizing CDK inhibitors. While CDK4/6 inhibitors are currently FDA-approved for the treatment of certain metastatic breast cancers, extensive research over the years has highlighted CDK inhibitor's efficacy in melanoma treatment. For instance, Sheppardd et al. reported that dysregulation of the p-16 cyclin D-CDK4/6-retinoblastorma protein pathway (CDK4 pathway) is found in 90% of melanoma cases. Activation of CDK leads to phosphorylation and inhibition of the retinoblastoma protein, thereby facilitating cell cycle transition from G1 to S phase, positioning CDK4 as a promising therapeutic target in melanoma [15]. The third-generation CDK4/6 inhibitors, such as ribociclib, abemaciclib, and palbociclib, have been evaluated as a single agent or in combination with BRAF±MEK inhibitors as first-line or subsequent therapies in patients. In a case report by Tang et al., melanoma was effectively controlled for over six months following the failure of chemotherapy or immunotherapy by employing CDK4/6 inhibition, thus underscoring the CDK4 pathway as a potential therapeutic target [16]. Research by Lelliott et al. has demonstrated that CDK4/6 inhibitors induced a T-cell intrinsic stem-like transcriptional signature, proceeding a clinical and immunological response to subsequence treatment with anti-ctla-4 and anti-pd1 therapy [17]. Incorporating CDK4/6 inhibitors into existing therapeutic protocols for melanoma holds promise in augmenting immune-mediated therapeutic responses to standard-of-care treatment. However, before releasing this case report, the patient in this case has yet to receive CDK4/6 inhibitors due to health insurance coverage and clinical protocols. Overcoming these challenges is essential to harness the full potential of CDK4/6 inhibitors in improving outcomes for melanoma patients.

This case underscores the real-time tracking capabilities of transcriptome analysis in monitoring gene expression and biomarkers, aiding clinicians in decision-making. Furthermore, transcriptome analysis can detect significant gene expression changes before phenotypic manifestation, offering a minimally invasive diagnostic approach. Overall, this case study highlights the potential of the transcriptome-based model as a novel approach in the clinical characterization and treatment of melanoma and other cancers.

## Conclusions

Malignant melanoma with brain metastasis has a high rate of mortality. Effective treatment for this advanced stage is limited, emphasizing the urgent need for innovative approaches to extend patient survival. Here, we presented the case of a 60-year-old male with melanoma and brain metastasis. By employing a transcriptome-based model to analyze samples of whole blood and tumor brain tissue, we identified unique gene expression patterns and enriched biological pathways in each sample type. Also, this model facilitates the linkage between enriched pathways/biomarkers to FDA-approved and investigated drugs, enabling personalized and precise treatment strategies. This case underscores the real-time tracking capabilities of transcriptome analysis in monitoring gene expression and biomarkers, aiding clinicians in decision-making, and highlights the potential of the transcriptome-based models as a novel approach in the clinical diagnosis and treatment of melanoma and other cancers.
